# A Comparison of Neural Networks and Center of Gravity in Muon Hit Position Estimation

**DOI:** 10.3390/e24111659

**Published:** 2022-11-15

**Authors:** Kadir Aktas, Madis Kiisk, Andrea Giammanco, Gholamreza Anbarjafari, Märt Mägi

**Affiliations:** 1 iCV Research Lab., Institute of Technology, University of Tartu, 51009 Tartu, Estonia; 2 Institute of Physics, University of Tartu, 51009 Tartu, Estonia; 3 GScan Ltd., Mäealuse 2/1, 12618 Tallinn, Estonia; 4 Centre for Cosmology, Particle Physics and Phenomenology (CP3), Université Catholique de Louvain, B-1348 Louvain la Neuve, Belgium; 5 Higher Education Institute, Yildiz Technical University, Istanbul 34349, Turkey

**Keywords:** cosmic-ray tomography, muon tomography, deep learning, particle detector, position estimation

## Abstract

The performance of cosmic-ray tomography systems is largely determined by their tracking accuracy. With conventional scintillation detector technology, good precision can be achieved with a small pitch between the elements of the detector array. Improving the resolution implies increasing the number of read-out channels, which in turn increases the complexity and cost of the tracking detectors. As an alternative to that, a scintillation plate detector coupled with multiple silicon photomultipliers could be used as a technically simple solution. In this paper, we present a comparison between two deep-learning-based methods and a conventional Center of Gravity (CoG) algorithm, used to calculate cosmic-ray muon hit positions on the plate detector using the signals from the photomultipliers. In this study, we generated a dataset of muon hits on a detector plate using the Monte Carlo simulation toolkit GEANT4. We demonstrate that two deep-learning-based methods outperform the conventional CoG algorithm by a significant margin. Our proposed algorithm, Fully Connected Network, produces a 0.72 mm average error measured in Euclidean distance between the actual and predicted hit coordinates, showing great improvement in comparison with CoG, which yields 1.41 mm on the same dataset. Additionally, we investigated the effects of different sensor configurations on performance.

## 1. Introduction

Cosmic-ray-based tomography systems generally consist of several plates which are positioned around the target material [1,2,3,4]. Such plates are position-sensitive particle detectors with the ability to detect the passage of electrically charged particles such as muons, therefore making it possible to learn about their trajectories. For example, ref. [2] discusses a system that positions three detector plates below and another three above the material of interest to obtain precise information about muon trajectories through the system. In many tomography systems, detector plates are composed of luminescent materials and silicon photomultipliers (SiPM). Thus, when a muon crosses the plate, luminescent material produces light that is collected by the SiPMs. Then, the signal amplitudes of all SiPMs are processed to estimate the location of muon hits.

There are different ways to process the sensor amplitudes to estimate the hit position, and conventional analytical methods, such as Center of Gravity (CoG), have been used for this purpose [5,6]. However, the low performance of such methods motivated researchers to look for alternative techniques. Aguiar et al. simulated a plate detector and used Anger logic for localization of the muon hits [7]. Recently, thanks to the latest technological developments, machine learning-based methods have become popular, in addition to the analytical methods for estimating the particle hit position. Their benefits such as better spatial resolution or faster processing capabilities are explored in various studies. Yonggang et al. implemented a multilayer perceptron (MLP) network with 2×8 neurons on an FPGA [8]. They showed that machine-learning-based methods produce promising results, while they can lead to a more computationally efficient estimation in real-time application. In [9], the authors use a similar network architecture as in [8]. However, they took a different approach to the data configuration and the network training. They split *x* and *y* axes into 7 intervals each. They first train a global network to predict the *x* and *y* positions roughly. Then, they use a sub-interval network to find an exact location. They trained 7 neural networks for *x* and 7 neural networks for *y* as sub-interval networks. In addition to MPL architecture, [10] used a convolutional neural network (CNN) to decode the gamma-ray interaction locations, achieving an average spatial resolution of 0.40 mm. They collected the light distribution as a 16-pixel image using the sensors placed at the edges of the plate. Then, they used a network that consists of a CNN layer with 200 filters of size 1 × 1 combined with a fully connected (FC) layer.

Hit positions of the muons are the most important data for cosmic-ray-based tomography systems, since the whole system is designed according to the trajectories of muons that are calculated using the hit positions. Due to this, the resolution of a hit position estimation carries the utmost importance. Improvement of the estimation method results in a better hit position estimation, consequently improving the imaging performance of the tomography system. Although there have been studies to enhance performance on this task, the literature is limited, and there is still a big research gap, especially in deep-learning-based methods. Studies about the performance of deep neural networks (DNN) in the hit position estimation task are very limited. Comparison of DNN and CoG, and DNN’s performance for different sensor configurations, are two topics to be explored. Therefore, in this study, we investigate how much one could increase the positional resolution by replacing CoG with deep neural networks. We present a fully connected architecture and a CNN-based architecture to evaluate the performances of different networks compared with CoG. Our results cover a comparison of resolution data. Optimization of physical detection systems is out of the scope of this study. There are three main contributions of this research:

- We present a simulated dataset of muon hit positions on a detector plate.
- We present two deep-learning-based methods for the muon hit position estimation task. We compare their performances with CoG. Additionally, we investigate the proposed method’s performance in depth.
- We evaluate the effects of different SiPM configurations on performance.

The rest of this paper is organized as follows: Section 2 describes the dataset. Section 3 explains the proposed method. Section 4 presents and discusses the experimental results. Finally, Section 5 concludes this work.

## 2. Dataset

We characterized the response of a muon detector based on a plastic scintillator slab to create our dataset. We used the Monte Carlo simulation package GEANT4 [11] version 10.5 to model the main parameters of the detectors. The parameters used in simulations are:

- The plastic scintillator is an EJ-200 with light output 10,000 photons/MeV.
- The plate surface area is 400 cm${}^{2}$ (20 cm × 20 cm), and the plate thickness is 10 mm.
- The SiPMs sizes are 6 mm × 6 mm. Spacing between SiPMs placed on the top surface is 20 mm. The spacing is chosen heuristically, considering our efforts to keep the sensor number as low as possible due to the cost.
- The detection efficiency of SiPMs is 40%.
- The reflective material used in simulations is diffusive reflection material with the surface covered with TiO${}_{2}$ paint (reflection efficiency 95%).

**Figure 1:**
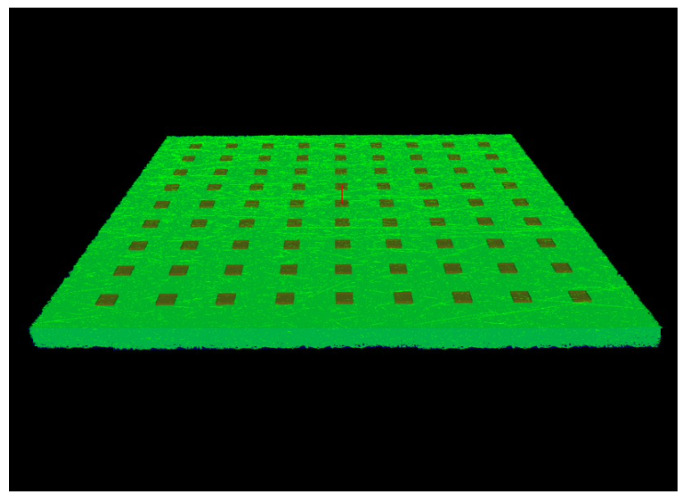
The simulated detector plate.

The simulated detector plate can be seen in Figure 1. In this figure, the red squares represent SiPMs, and the green surface represents the scintillator area. According to our setup, the SiPMs face the top of the detector plate. A symmetrical placement of SiPMs on both axes is followed as the plate is square-shaped.

**Figure 2:**
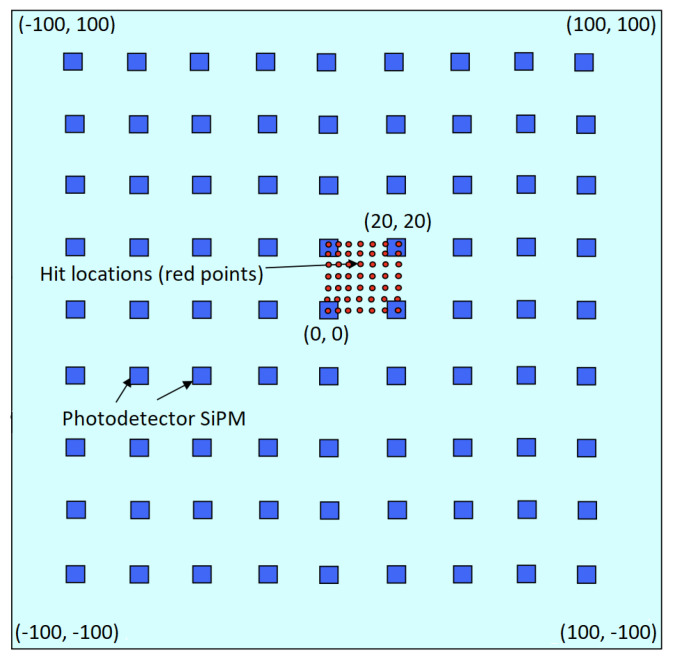
Simulated hit locations on the simulated plate, in mm.

We simulated muon hits only on one specific area, instead of the whole surface. Figure 2 shows the simulated hit locations as red points. We chose the hit locations only in this limited area to save computation time, as the system is periodically invariant by translation. Muons are emitted perpendicular to the detector surface in a grid with a step of 1 mm. Therefore, we created 441 different hit positions. For each hit position, we simulated around 10,000 hits. Our total simulated hit count added up to 4,416,335 (∼441 × 10,000). When a muon hits the detector plate, the scintillation light spreads in the plate and is detected by SiPMs. We saved the signal amplitudes from SiPMs and hit coordinates (*x*, *y*) for each hit. Thus, we created a dataset of hit coordinates and corresponding signal amplitudes. Sensors are placed in the middle of the plate from the side view. Therefore, when a muon hits the exact location of the sensor, it causes a light to be produced on the plate as usual. The sensor at the hit location ends up with the highest signal amplitude, since it is the closest to the source.

Finally, we created sub-sets of this dataset by discarding outer sensor signal amplitudes. For example, we obtained a sub-dataset for 64 (8×8) sensors by discarding the outer signal readings from the data. Following the same logic, we created datasets for 49 (7×7), 36 (6×6), 25 (5×5), and 16 (4×4) sensors, separately. This way, we could experiment with how different SiPM configurations affect performance. Since we obtained the sub-sets by cropping outer sensor amplitudes from the 81-sensor data, the hit locations and the parameters remain the same for the different sensor configurations.

**Figure 3:**
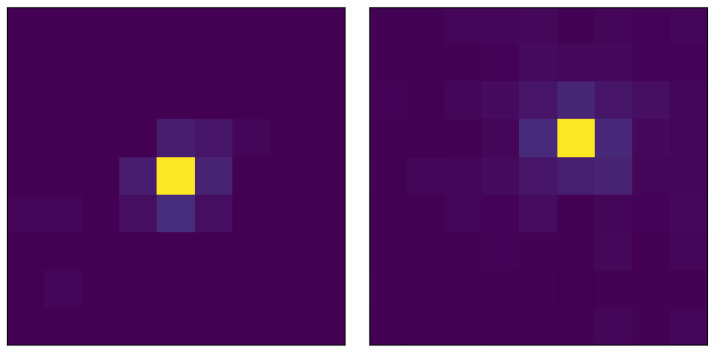
Samples of signal amplitudes for hits on (0, 0) and (20, 20) mm, respectively, where brighter color indicates a larger increase in amplitude.

It is possible to treat each signal amplitude as a pixel and show the illumination from a single-particle hit as an image. This way, we present the samples from (0, 0) and (20, 20) in Figure 3. From the samples, it can be seen that as the hit coordinate changes, the illumination output changes. To show an obvious change, we demonstrated outputs of hits on (0, 0) and (20, 20) coordinates. Higher signal amplitudes from sensors, i.e., brighter pixels, shift position according to the hit position.

## 3. Proposed Methods

In this paper, we propose two different methods to estimate the 2D hit position of muons on scintillation detector plates. We call them Fully Connected Network (FCN) and CNN. In previous studies, it has been proven that the neural networks can achieve good spatial resolution on particle hit position estimation on positron emission tomography (PET) systems [8,9,10]. Since we have a similar task, we decided to utilize neural networks in our methods as well.

### 3.1. Fully Connected Network

In previous research, a multilayer perceptron (MLP) has proved to be a successful structure in similar tasks [12,13]. Therefore, we based our FCN on an MLP network. Our network consists of an input layer, a batch normalization layer, a flattened layer, 2 fully connected layers with 256 and 32 neurons, and an output layer. A batch normalization layer is added to regularize the input. Then, the flattened layer converts the regularized input to the desired shape for fully connected layers. Later, the features are extracted, and a single-neuron output layer is used to obtain the estimated position coordinate.

In our study, we consider *x* and *y* coordinates of hits separately. So, instead of estimating them using a single model, we trained two different models, one FCN for *x* and another FCN for *y*, independently, since it is possible to train different networks for each coordinate at this point.

**Figure 4:**
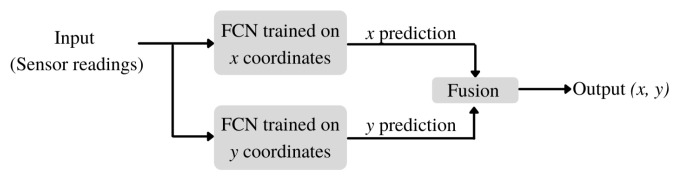
End-to-end pipeline.

The end-to-end structure of the proposed method can be seen in Figure 4. Firstly, signal amplitudes are passed to each model as input. Then, *x* and *y* coordinate predictions are obtained from the networks separately. Lastly, a fusion is performed to obtain the final *(x, y)* coordinate by combining both predictions as a single output.

### 3.2. Convolutional Neural Network

In scintillation detector plates, photosensors are used to detect and transfer muon hit data to a digital context. For example, in a setup with 9×9 (81) photosensors, the signal amplitude of each photosensor is transferred to the processor, and the data obtained can be considered as a 9×9 image. According to muon’s hit position, the shape of this 9×9 image changes (see Figure 3) because different hit positions cause different signal amplitudes on each sensor, since the illumination produced by the plate changes accordingly.

Convolutional layers in a deep neural network iterate through all the pixels in the input images to extract the features. Due to this, they proved to be very successful in various image analysis tasks [14,15,16,17,18]. Considering our input data, the task at hand can be approached as an image processing task, and the CNN can be used to catch the visual cues. The output of the convolutional layer for a pixel is calculated by the following formula [19]:

(1) $$\begin{array}{c} y_{i,j}=\sum_{m}\sum_{n}w_{m,n}x_{\left(i+a),(j+b\right)} \end{array}$$

where *x* is the input, *y* is the output of next layer, and *w* is the kernel.

Utilizing the convolutional layers, we developed a CNN-based architecture. This network includes an input layer, a batch normalization layer, two convolutional layers, and an output layer. A batch normalization layer is added for regularization as in the previous network. Then two CNN layers with 64 and 32 depths are used to extract the visual features. Kernels with sizes 3×3 are selected for these layers. Lastly, the output layer is added to reduce the network outcome to a single coordinate. In our experiments with the CNN, we simply replaced the FCN models in the pipeline (See Figure 4) with the CNN.

### 3.3. Training

We followed the same training process for all the sensor configurations. The only difference is the number of sensor readings used. The process of dataset preparation for the different sensor configurations is explained in Section 2. For the training, we split the data into 3 groups: training, validation, and testing by a ratio of 0.6, 0.2, and 0.2 of total data. The training and validation data (80% of total data) are used in training the model. On the other hand, test data (20% of total data) are not included in the training phase. Test data are only used to measure the performance of the model after the model is ready.

During the training, the model processes the data over and over again, and thus learns their characteristics in a certain number of iterations. In this regard, one full pass of the data is called an epoch. During an epoch, the model updates itself repeatedly after batches of data. The size of this batch and the number of epochs are configurable parameters in the training [20]. In our experiments, we used 100 epochs and a batch size of 32, as these values provided us with a proper learning process. However, since a model can converge quickly, we added an early stopping to prevent overfitting.

**Figure 5:**
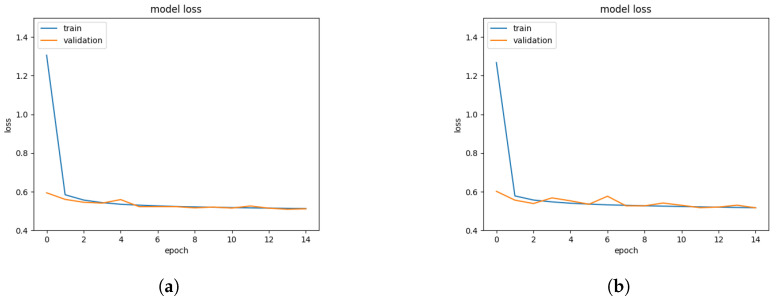
Learning curves for FCN trainings with 81-sensor data. (**a**) Curve for the model for *x* prediction. (**b**) Curve for the model for *y* prediction.

We also applied the Adam optimizer [21] with a learning rate of 0.001. We repeated the same training process for all the networks we trained. In Figure 5, you can see the training curves for two models, which are trained to predict *x* coordinates and *y* coordinates individually, using 81-sensor data. It can be seen that the models converge quickly, and overfitting is not observed during the training.

Mean squared error (MSE) is used as the loss function to measure the performance during the training:

(2) $$\begin{array}{c} MSE=\frac{1}{N}\sum_{i=1}^{N}{\left(x_{i}^{\prime }-x_{i}\right)}^{2} \end{array}$$

where *N* is the number of samples, *x* is the actual value, and *x${}^{\prime }$* is the predicted value.

## 4. Experimental Results and Discussion

Firstly, we compared FCN and CNN to see which model performs better. In this experiment, we used signal amplitudes from 81 sensors as the input data. The predicted hit coordinates are compared with the actual hit coordinates to obtain an error. We calculated the error by euclidean distance, and took an average of errors for all the predicted hits to obtain a single average error value as result:

(3) $$\begin{array}{c} Error=\frac{1}{N}\sum_{i=1}^{N}\sqrt{{\left(x_{i}^{\prime }-x_{i}\right)}^{2}+{\left(y_{i}^{\prime }-y_{i}\right)}^{2}} \end{array}$$

where *N* is the number of samples, *x* and *y* are the actual hit positions, and *x${}^{\prime }$* and *y${}^{\prime }$* are the predicted hit positions on the *x*-axis and *y*-axis, respectively.

Table 1 shows the performance of each network. Both networks achieve an error lower than 0.8 mm, however, FCN performs better by a small margin. We discuss that our CNN could not extract features that are more differentiating than the ones extracted by FCN. The CNN extracts the features by relating the pixels with the neighboring pixels thanks to the convolution operation iterated through the whole image. Therefore, we suspect that the image size is too small to utilize the full capability of the CNN because, in our case, the count of the pixels with a significant value is fairly low. Considering the results in Table 1, we chose FCN as our proposed method and continued the experiments using it.

Additionally, we experimented using different SiPM configurations. Initially, our dataset had signal amplitudes from 81 sensors. However, as explained in Section 2, we discarded signals from outer sensors and evaluated the effect of SiPM amount on performance. Considering the possible complexity of cosmic-ray tomography systems, being able to use fewer materials to obtain a competitive performance would decrease the cost and implementation complexity.

In Table 2, we present the performance of FCN for different sensor configurations. In the table, we can see that the performance is very close for 81, 36, and 25 sensors with average errors, respectively, 0.72 mm, 0.73 mm, and 0.75 mm. The first big jump in the average error is obtained when the sensor count is reduced to 16. From these results, we argue that the sensor amount does not necessarily need to be as high as possible. We can see that after 25 sensors, an increase in the sensor count does not necessarily improve the performance by a lot. For this reason, a trade-off should be carried out between the performance and the cost to find a satisfying spot. For our case, we find 25 sensors plausible, as we do not want to have more than triple the amount of sensors to obtain an increase of 0.03 mm in performance.

Another important point in Table 2 is the trend seen there. It is clear that, as there are more sensors, better performance is obtained. This is expected as more sensors mean a more precise estimate of the illumination caused by the hit, which in turn results in a more precise prediction of the position. However, we can also see that after some point, the increase in the number of sensors is not so effective. We explain this by the behavior of the light produced by the hit. Whenever a hit happens, the hit point becomes the source of the light. This means, as we go away from the hit position, signals become weaker. Eventually, the sensors on far positions have very small signal amplitudes, hence carrying little information. An example of the signal amplitudes for a particle hit on coordinates (0,0) is given below. As expected, the sensors around the hit position have the highest signal amplitudes, while the outer sensors become insignificant gradually. It should be noted that the average value of signal amplitudes is on the same level for different hit locations, although the pattern shifts around:

(4) $$\begin{array}{c} \left[\begin{array}{ccccccccc} 0 & 2 & 3 & 4 & 3 & 3 & 7 & 3 & 5\\ 1 & 4 & 7 & 6 & 7 & 4 & 2 & 3 & 2\\ 5 & 3 & 5 & 7 & 2 & 14 & 10 & 5 & 1\\ 2 & 3 & 10 & 6 & 12 & 22 & 8 & 6 & 4\\ 3 & 6 & 10 & 36 & 250 & 52 & 14 & 5 & 6\\ 7 & 7 & 10 & 46 & 132 & 56 & 13 & 12 & 4\\ 4 & 7 & 10 & 20 & 44 & 25 & 10 & 7 & 3\\ 5 & 4 & 9 & 7 & 3 & 13 & 9 & 7 & 8\\ 3 & 3 & 5 & 1 & 9 & 9 & 9 & 2 & 5 \end{array}\right] \end{array}$$

To have a detailed look into the performance, we also explored the results for *x* and *y* coordinates separately. For this purpose, in Table 3, we demonstrate the number of predicted coordinates and corresponding intervals for their errors. For example, 97.35% of *x* predictions fall in the 0–1.2 mm error interval. From the table, we can see that similar results are obtained for both models trained on the *x* and *y* coordinates, separately. Similar performances for both models are expected as our setup is symmetrical in sensor placement. Additionally, only 0.01% and 0.03% of the estimations have an error over 6 mm. This shows that the outliers are negligible.

Finally, to evaluate the performance against a commonly used conventional method, we compare our results with those obtained with CoG:

(5) $$\begin{array}{c} x=\sum_{i=1}^{N}x_{i}\cdot A_{i}{\left(\sum_{i=1}^{N}A_{i}\right)}^{-1} \end{array}$$

(6) $$\begin{array}{c} y=\sum_{i=1}^{N}y_{i}\cdot A_{i}{\left(\sum_{i=1}^{N}A_{i}\right)}^{-1} \end{array}$$

where $x_{i},y_{i}$ are the coordinates on the detector plate, $A_{i}$ is the corresponding amplitude, and x,y are the CoG positions.

As it can be seen in Table 4, our deep-learning-based methods produce significantly better results compared with CoG on average. One of the benefits of using analytical methods such as CoG is that they do not require a big dataset to learn, unlike a deep learning model. However, in our case, the dataset is viable. Therefore a significant gain in performance is definitely preferable.

**Figure 6:**
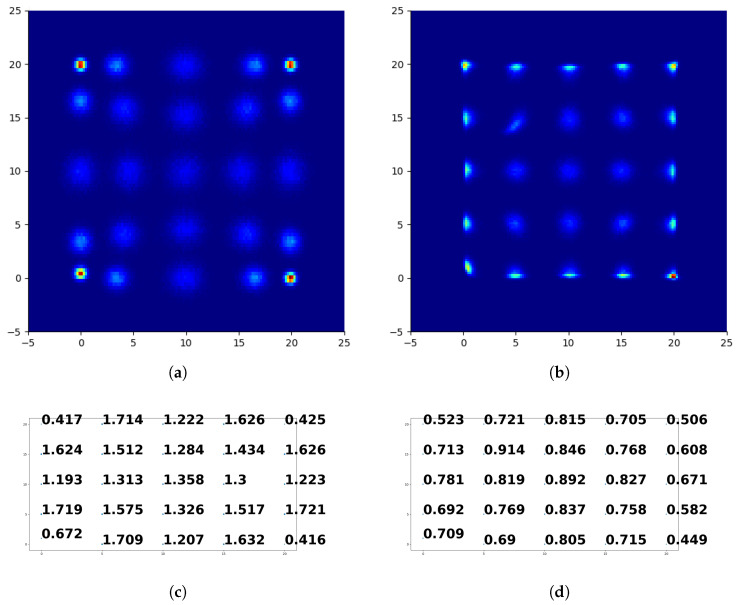
Comparison of estimations for CoG and FCN for some hit locations. (**a**) Distribution of CoG estimations for the hits on the selected locations, in mm. (**b**) Distribution of FCN estimations for the hits on the selected locations, in mm. (**c**) CoG average errors per hit location with respect to Figure 6a. (**d**) FCN average errors per hit location with respect to Figure 6b.

We visualize hit location estimations for some coordinates, i.e., with a 5 mm step, in Figure 6 to have a more detailed comparison between CoG and FCN. The area in this figure represents the red dotted area shown in Figure 2. The dark blue area represents the coordinates where there are no estimations, and the transition from light blue to red represents the increasing number of hits estimated on the respective coordinates. We show only twenty-five hit locations in this display. We did not include all the hit locations used in the experiment here to be able to present the results clearly. However, the results follow the same trend for the other hit locations as well. We present average errors obtained for these locations together with the estimations. Due to a technical issue in our simulation, some of the data at the (0,0) location have an uncharacteristic distribution of signal amplitudes. To avoid biases from this spurious effect, we utilize hits at (0,1) instead of (0,0) to have better clarity on the results, as the results follow the same trend.

For CoG, in Figure 6a,c, hits at the sensor locations (0,1), (0,20), (20,0), and (20,20) are estimated more accurately compared with other locations. CoG weighs the closer sensors the most because of their high signal amplitudes, so this behavior is expected. The performance decreases as the hit locations move away from the sensors. Moreover, it can be seen that the estimations around the sensors have a bias towards the sensors, which causes a lower performance around the sensors. This creates a certain pattern for CoG estimations, which is expected due to the nature of the algorithm. On the other hand, FCN estimations follow a different pattern. By comparing Figure 6c,d, we can see that the proposed method obtains smaller errors than CoG for all hit locations except (0,1), (0,20), (20,0), and (20,20). For these particular locations, however, the average errors are similar between the methods. Compared with CoG, the estimations for FCN are concentrated around the actual hit locations more since the errors are smaller.

**Figure 7:**
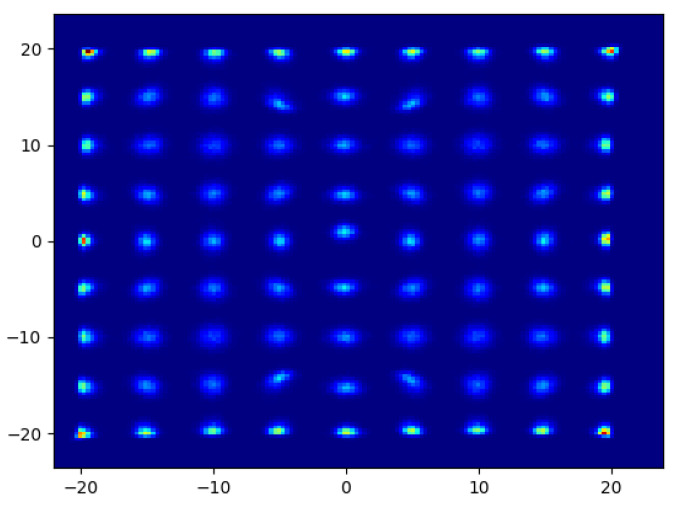
Distribution of FCN estimations for the hits on the extended area which covers the middle part of the plate, in mm.

An important point to consider about FCN is a generalization. In Figure 6b, the estimations at the edges have sharper shapes compared with the inner ones. We argue that this is a result of the characterization of the restricted data used during the training by the model. The model learns that the hits are restricted in this particular area, so the estimations are limited to the same area. However, this issue can be overcome by using a dataset that includes hits from the whole area targeted. To demonstrate that, we simply multiplied the current data symmetrically along the axes to cover an extended area and trained another model using the extended data. The multiplication of the data is performed by first exploiting the symmetry of the signal amplitudes and corresponding hit locations on the *y*-axis, then exploiting their symmetry on the *x*-axis. The results of this experiment can be seen in Figure 7. The average error stays the same with the previous surface at 0.72 mm. A similar error is expected since the data are symmetrical. In the figure, the previously restricted locations such as hits along the axes are now estimated without a restriction. This experiment shows that the method can cover the whole plate if the data are generated accordingly. In that case, the restricted area would be the plate itself, which is a desirable outcome.

## 5. Conclusions

Cosmic-ray tomography systems utilize various detector plates, and their imaging performances heavily depend on the particle hit position estimation on those plates. A precise estimation of the hit positions can increase the produced image’s quality, and vice versa, a bad estimation may cause low-quality or even meaningless images. Therefore, a successful position estimation carries the utmost importance in such systems. Traditionally, analytical methods such as Center of Gravity have been used for particle hit position estimation. These methods do not require a big dataset, on the other hand, their performances are relatively poor, as they do not exploit the full information available in a detector layer. Thus, research has been conducted on alternative methods, such as deep learning techniques. So far, deep-learning-based methods have produced promising results for hit position estimation, but there is still a research gap.

In this paper, we investigated the performance of two deep-learning-based networks, i.e., FCN and CNN on the muon hit position estimation task. We collected a muon hits dataset produced by GEANT4 and obtained an average error of 0.72 mm using FCN with 81-sensor data. This is a significant improvement over an analytical method such as CoG. We also investigated the effects of different sensor configurations in our setup. We saw that 25 sensors produce results almost as good as 81 sensors. As part of future work, the effect of different intervals on hit positions simulated can be investigated to improve the performance further. Moreover, the dataset can be extended to cover a larger plate area and include varying hit angles. Additionally, our plans involve building a realistic experimental setup to compare the results of this study with real device data. 

## Figures and Tables

**Table 1:** Comparison of FCN and CNN.

Method	Average Error
FCN	0.72 mm
CNN	0.79 mm

**Table 2:** Performance of FCN for different SiPM amounts.

Number of Sensors	Average Error
81 (9 × 9)	0.72 mm
36 (6 × 6)	0.73 mm
25 (5 × 5)	0.75 mm
16 (4 × 4)	0.84 mm
9 (3 × 3)	1.08 mm

**Table 3:** *x* and *y* predictions with the corresponding error intervals.

Error Interval	*x* Predictions	*y* Predictions
0–1.2 mm	97.35 %	97.28 %
1.2–2 mm	2.0 %	2.11 %
2–4 mm	0.59 %	0.53 %
4–6 mm	0.05 %	0.05 %
6 mm and higher	0.01 %	0.03 %

**Table 4:** Comparison of average errors for FCN, CNN, and CoG using the 81-sensor data.

Method	Average Error
FCN	0.72 mm
CNN	0.79 mm
CoG	1.41 mm

## Data Availability

The dataset used in this paper is available from the authors upon request.
